# Role of Hypothalamic Melanocortin System in Adaptation of Food Intake to Food Protein Increase in Mice

**DOI:** 10.1371/journal.pone.0019107

**Published:** 2011-04-22

**Authors:** Bruno Pillot, Céline Duraffourd, Martine Bégeot, Aurélie Joly, Serge Luquet, Isabelle Houberdon, Danielle Naville, Michèle Vigier, Amandine Gautier-Stein, Christophe Magnan, Gilles Mithieux

**Affiliations:** 1 Institut National de la Santé et de la Recherche Médicale, U855, Lyon, France; 2 Université de Lyon, Lyon, France; 3 Université Lyon I, Villeurbanne, France; 4 Université Paris Diderot, Unit of Functional and Adaptive Biology (EAC4413), Paris, France; University of Camerino, Italy

## Abstract

The hypothalamic melanocortin system—the melanocortin receptor of type 4 (MC4R) and its ligands: α-melanin-stimulating hormone (α-MSH, agonist, inducing hypophagia), and agouti-related protein (AgRP, antagonist, inducing hyperphagia)—is considered to play a central role in the control of food intake. We tested its implication in the mediation of the hunger-curbing effects of protein-enriched diets (PED) in mice. Whereas there was a 20% decrease in food intake in mice fed on the PED, compared to mice fed on an isocaloric starch-enriched diet, there was a paradoxical decrease in expression of the hypothalamic proopiomelanocortin gene, precursor of α-MSH, and increase in expression of the gene encoding AgRP. The hypophagia effect of PED took place in mice with invalidation of either MC4R or POMC, and was even strengthened in mice with ablation of the AgRP-expressing neurons. These data strongly suggest that the hypothalamic melanocortin system does not mediate the hunger-curbing effects induced by changes in the macronutrient composition of food. Rather, the role of this system might be to defend the body against the variations in food intake generated by the nutritional environment.

## Introduction

The worldwide expression of obesity and type 2 diabetes makes more and more intriguing a better understanding of the mechanisms regulating food intake [1], [2]. The brain, more particularly the hypothalamus, plays a central role in this control. The hypothalamus integrates circulating signals of hunger and satiety together with nutrient-derived signals that reflect the availably of energy stores of the body. This allows the brain to adapt energy intake and energy expenditure to the actual requirement of the body [3].

Indeed, insulin or leptin, two major hunger-suppressing hormones, are able to cross the blood brain barrier at the level of the hypothalamus and surfeit energy intake by generating anorectic signaling upon binding to their receptor expressed in neurons of the arcuate nucleus [1], [3], [4]. Conversely, ghrelin has a positive action on food intake that is mediated, at least in part, via the binding to ghrelin receptor located on arcuate neurons [5], [6]. In addition, a number of gastrointestinal hormones, released after the meal, have the capacity to promote satiation and/or satiety. Cholecystokinin, glucagon-like peptide 1 or PYY belong to these gastrointestinal hormones capable of decreasing hunger [7], [8], [9]. Regarding these hormones, experiments of vagotomy have strongly suggested that they could be sensed peripherally, and transmitted to the brain via the gastrointestinal nervous system [9], [10], [11], [12]. However, in all cases, the hypothalamus constitutes the ultimate site of integration of both centrally- and peripherally-mediated hormonal mechanisms of control of food intake [1], [2], [3].

Within the hypothalamus, the melanocortin system is a hormone-receptor system that plays a crucial role to translate the sensing of hunger-modulating hormones into changes in the sensation of hunger and satiety. MC receptors are widely expressed throughout the hypothalamus [13], [14] and, among the MC receptors, the MC3R and MC4R are particularly involved in energy balance regulation. α-melanocortin-stimulating hormone (α-MSH), a postranslational product of the Pro-opiomelanocortin (POMC) gene, binds to the MC receptor on key structures in the central nervous system and triggers an anorectic signal [15]. POMC gene is expressed in neurons located in the arcuate nucleus. In parallel, other neurons of the arcuate nucleus express the neuropeptide Y (NPY) and the agouti-related protein (AgRP), an inverse agonist of MCR that prevents α-MSH binding onto MC3R and MC4R. Consequently, these neurons activate hunger by suppressing the α-MSH action. That the melanocortin system (MCS) is central in the control of the sensation of hunger and satiety has been ascertained from genetic studies in humans and mice. Hence, MC4R-Ko mice rapidly develop a dramatic obese phenotype consecutive to hyperphagia [16], while mutations in the MC4R gene have been associated to morbid obesity in humans [17]. Moreover, transgenic mice over-expressing AgRP develop early hyperphagia and obesity [18].

Whereas the peripheral and central control of food intake has been widely studied, the central mechanism that modulates the response to food quality and composition are scarce. A better knowledge of this mechanism, however, could provide a rationale for nutritional manipulation as a potential strategy to prevent and to treat obesity and diabetes. Indeed, the composition of the diet may be deleterious, causing obesity and insulin resistance, which is the case for western diets rich in lipids and in sweet [19], [20]. On the contrary, it could be beneficial, which is the case for protein-enriched diets (PED), initiating satiety phenomena [21], [22] and amelioration of glucose control in type 2 diabetic patients [23]. The MCS stands as a likely candidate to mediate the behavioral, hormonal and metabolic consequences of nutritional manipulation [24]. However, in a previous study pertaining to the regulation of the expression of the hypothalamic MCS by a high fat-diet, we unexpectedly observed an increase in the POMC to AgRP ratio, whilst mice fed this type of regimen exhibited hyperphagia and obesity [25]. This has suggested that the MCS might combat, rather than mediate, the changes in food intake induced by the diet. However, the interpretation of the data from high-fat diets is not unequivocal. Whereas the animals increased their intake in terms of calories ingested, they slightly decreased it in terms of weight of food ingested (due to the high caloric density of HF-HS diet compared to control chow).

To better understand the role of the MCS in mediating the effect of diet composition and quality onto energy balance, we have dissected out the implication of several cellular and molecular components of the MC system in the control of food intake by nutrients. We studied here the changes in MCS-related gene expression, body weight and food intake initiated by a high-protein (HP) diet. The diet used was balanced for increased proportion of protein by decreasing the carbohydrate content. Caloric density was thus identical to control chow diet.

Furthermore, to question the causal role of the MCS, we studied the effect of HP diet in mice with deletion of the components of the system.

## Materials and Methods

### Animals

#### Wild-type mice

Two mouse strains were used as control: C57Bl/6 and 129Sv. For each strain, four weeks-old male mice were purchased from Harlan (Gannat, France) and housed at 21°C with normal light/dark cycle and free access to water and food. After one week acclimatization and feeding with A04 standard chow from SAFE (Augy, France), mice were randomized and divided in two groups, with free access to either starch-enriched diet (SED: 16% protein from soybean and fish, 55% starch glucose, 5% lipids, 6% mineral salts, 5% cellulose, 1% vitamins, 12% water, by weight) or a protein-enriched diet (PED: 54% protein from casein-soybean, 17% starch glucose, 5% lipids, 6% mineral salts, 5% cellulose, 1% vitamins, 12% water) from SAFE. Body weight and food intake were continuously recorded.

#### Gene invalidated mice

Two different knock-out mouse models were used. The first model, obtained from Jackson Laboratories (Bar Harbour, Maine, USA), was the loxTB*MC4r* mice developed by Balthasar et al. [26], that do not express MC4R and display morbid obesity. They were bred on a mixed C57Bl/6 and 129Sv background. The second model, POMC-deficient, was developed on a 129SV background by Challis et al. [27] and was obtained directly from this laboratory. As the homozygous POMC^−/−^ mice are deprived of all POMC-derived peptides, they must be supplied with corticosterone-supplemented drinking water (25 µg/mL final concentration). Heterozygous mice were mated and female mice were supplied with corticosterone during the last week of gestation and the whole lactation period. Homozygous mice were supplied with corticosterone from weaning and during the whole experiment. Those transgenic 5-week old mice were also divided in two groups fed either with SED or the same PED than wild-type mice. Food intake and body weight were recorded as for wild-type mice.

#### Production of mice lacking NPY/AgRP neurons

Heterozygous Agrp^DTR/+^ mice with the human diphtheria toxin receptor (heparin-binding epidermal growth factor) targeted to Agrp locus have been described [28], [29]. Briefly, a cassette containing the human DTR open reading frame was placed upstream of the initiation codon of Agrp in the first exon. Agrp^DTR/+^ express, therefore, the hDTR onto NPY/AgRP neurons, which allows the specific depletion of NPY/AgRP neurons by peripheral injection of DT toxin. For the experiments, homozygous Agrp^DTR/DTR^ males (on mixed 129/Sv×C57Bl/6 genetic background) were bred with C57Bl/6 wild-type, female Agrp^+/+^ mice, such that all the offspring would be Agrp^DTR/+^ heterozygotes. Litters born at the same time were either injected within 2–5 days after birth with DT (75 ng in 20 µl saline) or untreated (naïve). Mice were housed at 20–22°C with a 12 hr light/12 hr dark cycle and provided with standard mouse chow diet (A03 Safe diets 3,2 Kcal/g), unless otherwise indicated. The extent of NPY/AgRP neurons depletion was assessed by the lack of orexigenic response to peripherally injected ghrelin in mice lacking NPY/AgRP neurons. Prior to the feeding experiment the groups “naïve” and “treated” were individually housed at least 2 months prior to the experiment. During food intake measurements, body weight and food intake were monitored daily, then mice were switched to a PED, and maintained on this diet for 6 days. By the end of the 6 days, mice were switched back to SED.

All animal care and experimental procedures were approved by the animal ethics committee of the university of Lyon.

### Real-time quantitative RT-PCR analysis

After 4 days of SED or PED, respectively, 5 weeks-oldC57Bl/6 mice were killed by cervical dislocation after a 6-hour fasting period. Brain was removed. The whole hypothalamus was rapidly dissected out and frozen in liquid nitrogen. The limits of the hypothalamus for dissection were the optic chiasma at the anterior border, the mammillary bodies at the posterior border and, on both lateral sides, the hypothalamic sulci. The tissue was finally cut dorsally at 2 mm from the ventral face.Total RNA was extracted with the TRIzol Reagent (Invitrogen). The level of target mRNAs was measured by RT followed by real-time PCR. First-strand cDNAs were synthesised from 500 ng total RNA using M-MLV reverse transcriptase RNAse H minus (Promega, France) and oligo(dT) primers. PCR was realized with Master SYBR Green 1 Mixture (Roche Diagnostics, Germany) with specific primers : 5′-TCTCTATGTCCACATGTTCCTG-3′ (sens) and 5′-GGGGCCCAGCAGACAACAAAG-3′ (non sense) for MC4-R, 5′-CTCAAGAAGACAACTGCAGAC-3′ (sense) and 5′-TGAAGAAGCGGCAGTAGCAC-3′ (non sense) for AgRP, 5′-ATGCCGAGATTCTGCTACAGTCG-3′ (sense) and 5′-TTCATCTCCGTTGCCAGGAAACAC-3′ (non sense) for POMC and 5′-TTCCAGTATGACTCCACTCACG-3′ (sense) and 5′-AGACTCCACGACATACTCAGCA-3′ (non sense) for GAPDH. Quantitative PCR is realized using a light-cycler (Roche Diagnostics, Germany). A standard curve was systematically generated with different amounts of purified target cDNA, and each assay was performed in duplicate. GAPDH transcript was used as a reference and results are expressed by a ratio relative to GAPDH expression.

### Plasma hormone measurements

Plasma insulin and leptin concentrations were determined using commercially available assay kits (from Crystalchem, Chicago, USA and AbCys, PAris, France, respectively).

## Results and Discussion

**Figure 1:**
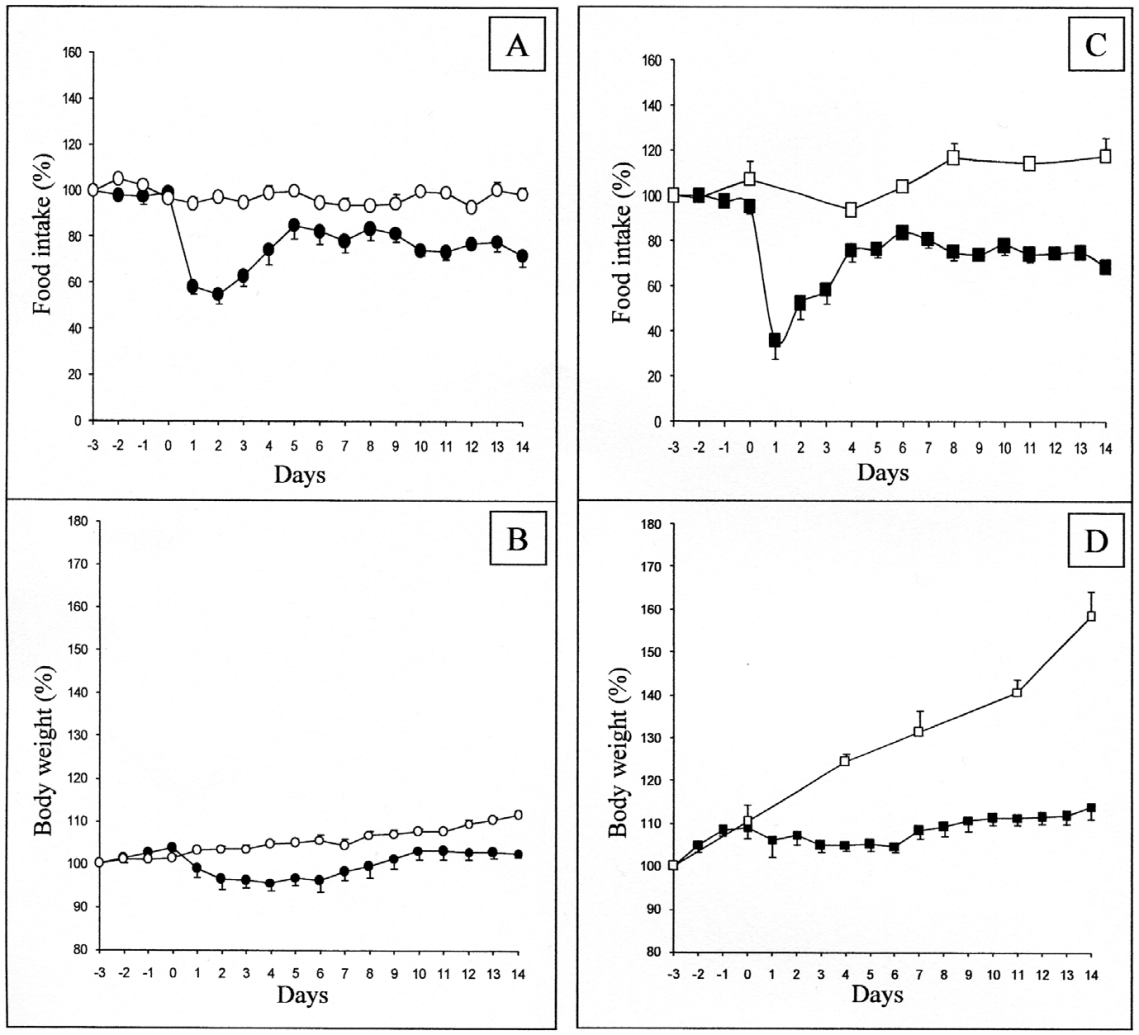
Effect of PED on food intake and body weight in C57bl6J and MC4-R KO mice. Food intake (panels A,C) and body weight (B,D) were measured in control mice (circles) and in MC4R-KO mice (squares), fed either on a starch enriched diet (SED, open forms) or on a protein enriched diet (PED, filled forms). PED was first given from “day 0”, mice being previously fed on the SED. For each point determination, n = 8 mice were studied. The data are expressed as means+/−SEM. The results have been reported to a reference value, calculated as the average of 3 prior consecutive days of SED (from days −6 to −4). The results are expressed as % of this reference. 100% represented 0.5 and 0.73 cal/d/g body weight in C57bl6J and MC4-R KO mice, respectively. All data from day 1 are different from day 0 for PED feeding (p<0.05, Student's t test for paired data), in both panel A and C.

It is well established that regimens enriched in protein decrease hunger sensations and subsequent food consumption in rats and humans [21], [22], [30], [31], [32]. In rats, after a transient period of some days of habituation to the HP food, the steady daily food intake was blunted by about 20% for the next two weeks [33]. When C57B6/J mice were given a HP diet, they markedly decreased their food intake the 2 first days (by about 40%). Then, they re-increased their food intake to about 80% of their previous intake with the iso-caloric chow diet (fig. 1A). This was comparable to the rat response to the HP diet [33]. As a consequence of diminished food intake, C57B6/j mice attenuated their growth rate upon HP diet, exhibiting a transient decrease in body weight followed by a phase of stabilization (Fig. 1B).

**Figure 2:**
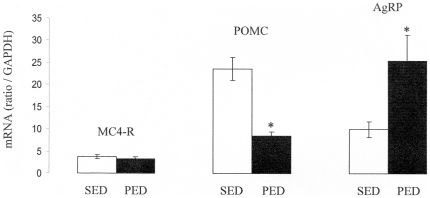
Hypothalamic contents in MC4-R, POMC and AgRPmRNAs. Determinations by quantitative RT-PCR. The relative mRNA contents were measured in hypothalamus of five-weeks-old C57bl6J mice after 4 days of diet of PED compared to SED fed mice, and reported to a GAPDH mRNA as a standard. *, p<0,05 (t test for unpaired samples).

Next, we analyzed the hypothalamic content of mRNAs encoding the MC4R, POMC and AgRP in mice fed on the HP diet, compared to their counterparts fed on the standard chow diet. There was no difference induced by the PED in the MC4R mRNA content (Fig. 2) whereas there was a marked decrease in the POMC mRNA content, by about 2.3 times (Fig. 2), and a marked 2.5 fold increase in the AgRP mRNA content (Fig. 2). This represented an increase by about 7 times of the AgRP to POMC ratio. In the same time, the animals were hypophagic. The analyses reported in Fig. 2 were performed after 4 days of protein regimen. One could argue that the AgRP to POMC expression change could be representative of an early phenomenon, aimed at compensating the early suppression of food intake consecutive to the switch on a novel diet. Therefore, we performed comparable analyses by 12 and 30 days after the switch on the protein diet, i.e. at a time of steady food intake. The results were comparable, revealing increases in AgRP and decreases in POMC expression (data not shown). There was, however, an attenuation of the changes with regards to the situation at 4 days, since the ratio of AgRP to POMC was increased by 3.3 times and 2.4 times, at 12 and 30 days after switching on the protein diet, respectively (results from 9 rats per group at each time, p<0.001 in each case). To question whether these changes could be related to a change in the hormonal status, we assayed plasma insulin and leptin after switching on the PED. There was no change in either plasma insulin or leptin under conditions of steady food intake, after the switch on PED: insulin: 0.82+/−0.12 vs 0.87+/−0.18 ng/mL in SED- and PED-fed mice, respectively (NS); leptin: 0.77 +/−0.23 vs 0.66+/−0.21 ng/mL in SED- and PED-fed mice, respectively (NS).

These data strongly suggested that protein-induced hypophagia was not mediated by an enhanced activity of the MCS, but rather support the hypothesis according to which a decreased MCS tonus is actively triggered to counteract the hypophagia effect, in order to maintaining the former food intake. Interestingly, this inference is in keeping with previous data from our group, and others, that illustrated a negative action of high-fat diets onto AgRP mRNA expression and the AgRP/POMC ratio, with a concomitant hyperphagia [25], [28], [29]. These previous data were not unequivocal, however, because of the high caloric density of HF diets (see introduction). Moreover, as previously acknowledged (Gout et al, 2008), other studies reported opposite results, which has suggested that the changes in AgRP/POMC could at least in part depend on physiological conditions related to the mouse strain, age, or time on the HF diet. We provide here stronger arguments in support of the aforementioned hypothesis. Indeed, because the HP diet and the control diet exhibited similar caloric densities, the changes in food intake were similar, that they were expressed as calories ingested by g of body weight per day, or as g of food ingested/g/day. It is noteworthy that the HP-initiated changes were specific of AgRP and POMC. There was no significant change in the expression of NPY (an orexigenic neuropeptide co-expressed with AgRP), induced by protein dieting (data not shown). We also analyzed the mRNA levels of some orexigenic neuropeptides expressed in the lateral hypothalamus: hypocretin (Hcrt) and pro-melanin-concentration hormone (Pmch, i.e. the precursor of melanin-concentrating hormone). There was no alteration in the mRNA levels of either neuropeptide induced by the protein diet (data not shown).

**Figure 3:**
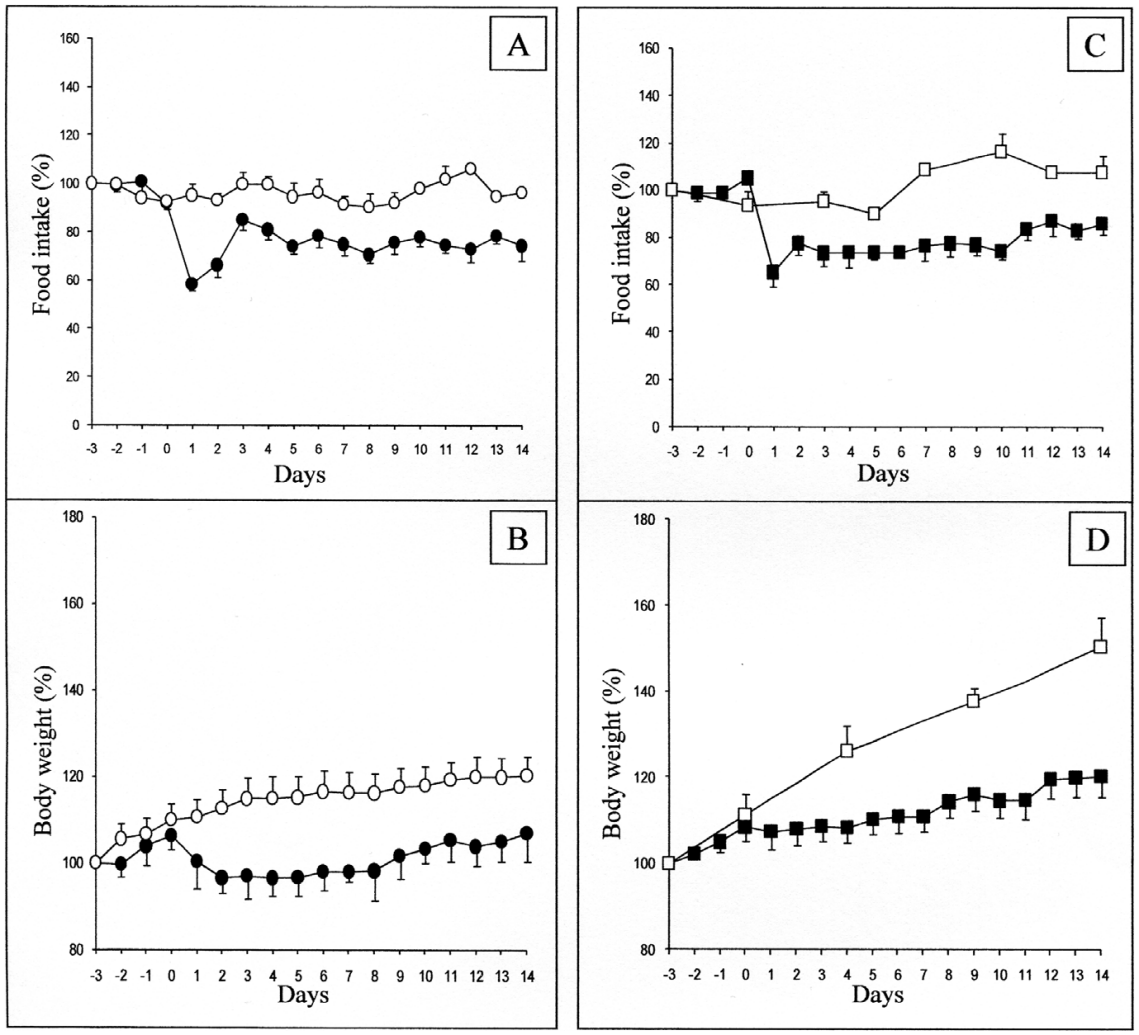
Effect of PED on food intake and body weight in SV129 and POMC KO mice. Food intake (A, C) and body weight (B, D) were measured in wild-type mice (circles) and in POMC-KO mice (squares), fed either on a starch enriched diet (SED, open forms) or on a protein enriched diet (PED, filled forms). Expression and presentation of data are as described in the legend of Fig. 1 (n = 8 mice studied). 100% represented 0.63 and 0.69 cal/d/g body weight in 129SV and POMC KO mice, respectively. All data from day 1 are different from day 0 for PED feeding (Student's t test for paired data), in both panel A and C.

**Figure 4:**
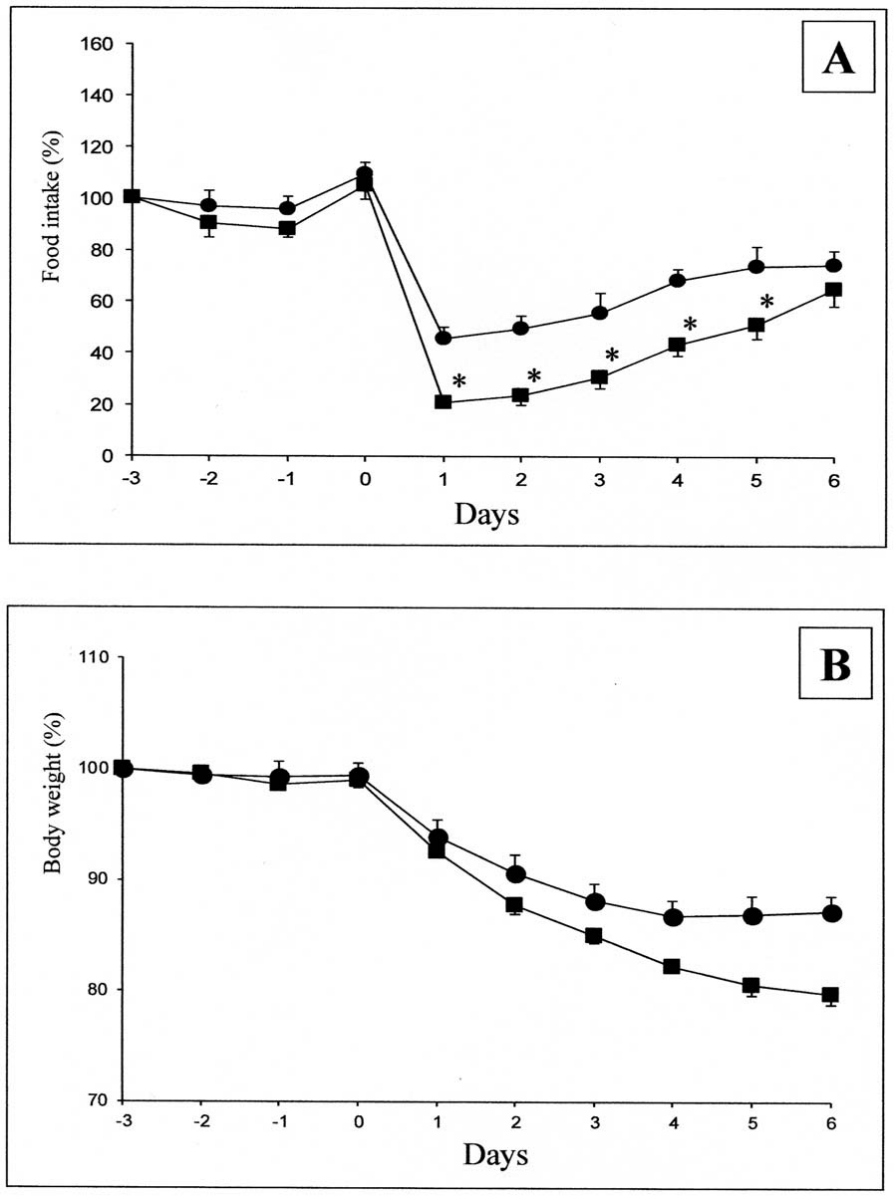
Effect of PED on food intake and body weight in SV129 and NPY/AgRP-neurons depleted mice. Food intake (A) and body weight (B) were measured in AgRP^DTR^ naive mice (circles) and in AgRP^DTR^ mice treated as pups (squares). Mice were fed from “day 0” on the PED diet. For each point determination, n = 6 mice were studied. At day 6, because AgRP^DTR^ treated mice still lost weight, PED feeding was stopped and mice were switched back on the control chow diet (SED). Body weight variation (B) is expressed in % of initial body weight and food intake (A) as % of reference food intake, as described in the legend of Fig. 1. All data from day 1 are different from day 0 (Student's t test for paired data) in panel A. *, different from wild-type mice (Student's t test for unpaired data).

To question more in depth the role of the MCS in the changes in food intake initiated by food quality changes, we studied the effect of HP diet in mice with deletion of the components of the MCS. MC4R-KO mice, backcrossed on a C57B6/J genetic background, like C57B6/J control mice, depressed their food intake when switched in the HP diet (Fig. 1C). The further evolution of food intake was very comparable to that in control mice. Similarly, there was a marked attenuation of their growth rate upon HP dieting (Fig. 1D). This strongly suggested that MC4R was not mandatory for the hypophagia effect induced by nutritional protein. Next, we questioned the effect of HP diet in mice with deletion of the POMC gene. Because these mice were backcrossed on a 129SV genetic background, and required a corticosterone treatment to prevent any deficiency in the corticotrope axis, we studied in parallel the effect of the HP diet in control 129SV treated with the same daily dose of corticosterone. Whereas the initial blunting of food intake upon switching on the HP diet was less marked than in the C57B6/J background, the steady state suppression of food intake about −20%) was very similar in both the control and the POMC-KO mice (Fig. 3A and C). In line with the suppression of food intake, there was a comparable attenuation of the growth rate of 129SV and POMC-KO mice upon feeding on the HP diet (Fig. 3B and D). This result strongly suggested that the POMC gene, precursor of α-MSH, was not essential for the hunger-suppressing effect of food protein diet. Lastly, we studied the effect of the HP diet in mice with deletion of the arcuate NPY/AgRP neurons. The strategy of deletion involves the expression of the receptor to the diphtheria toxin under the control of the AgRP gene promoter. Heterozygous AgRP^DTR/+^ mice were treated (AgRP^DTR/+^-injected), or not (AgRP^DTR/+^-naive), a few days after birth with the diphtheria toxin. Under these conditions of early deletion of the NPY/AgRP-expressing neurons, AgRP^DTR/+^-injected mice further exhibited a normal food intake under standard chow diet, as previously described [34], [35]. Upon switching onto protein diet, there was an early drop in food intake in AgRP^DTR/+^-naive followed by a progressive return to a food intake minored by about 20% (Fig. 4A). This drop was very similar to that observed in C57B6/J mice (compare with Fig. 1A). Interestingly, the drop observed in AgRP^DTR/+^-injected mice was significantly stronger than that in their littermates. It is noteworthy that such a difference was not observed in the changes in food intake promoted by protein diet in either MC4R^−/−^ or POMC^−/−^ mice, compared to their respective controls (see Fig. 1 and Fig. 3). Because these mice continuously lost weight, we stopped PED feeding to switch back on standard chow, when weight loss was beyond 20% of their starting body weight (Fig. 4B). These data suggest again that specific inhibition of NPY/AgRP neurons was not mandatory for the hypophagia induced by nutritional protein. Moreover, because the hypophagia effect appeared stronger in mice lacking the NPY/AgRP than in controls, this might suggest that NPY/AgRP-expressing neurons are important in a defense process to maintaining food intake and body weight during the hypophagic phase initiated by protein diet. This is in line with the fact that hypothalamic AgRP content and AgRP to POMC increased ratio is a key feature associated to the hypophagic response to protein feeding in the normal situation (results of Fig. 2). This observation, thus, further support our hypothesis. It must be noted that AgRP is still able to enhance food intake in mice invalidated for both MC3R and MC4R, which has suggested that AgRP could have an effect independent of its antagonist role towards both MCRs (see [36] for a recent review). Thus, the hypothesis that AgRP might interfere in mechanisms independent of MC3R and MC4R constitutes an additional attractive rationale.

Taken together, the data presented herein strongly suggest that activation of the satiety MCS is not mandatory for the inhibition of food intake induced by protein diet. This comes in discrepancy with the compelling body of arguments which have established that hypothalamic MCS is one of the main anorectic signals, through which most satiety actions are believed to occur [3], [37]. It is noteworthy to recall here that there is no change in plasma insulin or leptin concentrations, which could account for the AgRP/POMC changes observed on protein-diet (see above). Moreover, our results also suggest that inhibition of α-MSH-induced satiety signals is a likely mechanism involved to defend food intake and body weight against variations in hunger initiated by environmental nutritional changes, such as the variable availability in different sources of macronutrients. In conclusion, even if the changes in hunger and satiety depending on nutrients remain to be fully understood, the present study adds a significant piece in the understanding of the central mechanisms of control of energy homeostasis.

## References

[1] Flier JS (2004). Obesity Wars: Molecular Progress Review. Confronts an Expanding Epidemic.. Cell.

[2] Friedman MI (2000). Obesity in the new millennium.. Nature.

[3] Schwartz MW, Morton GJ (2002). Obesity: keeping hunger at bay.. Nature.

[4] Banks WR, Kastin AJ, Huang W, Jaspan JB, Maness LM (1996). Leptin enters the brain by a saturable system independent of insulin.. Peptides.

[5] kojima M, Hosoda H, Date Y, Nakazato M, Matsuo H (1999). Ghrelin is a growth-hormone-releasing acylated peptide from stomach.. Nature.

[6] Chen HY, Trumbauer ME, Chen AS, Weingarth DT, Adams JR (2004). Orexigenic action of peripheral ghrelin is mediated by neuropeptide Y and agouti-related protein.. Endocrinology.

[7] Moran TH (2000). Cholecystokinin and satiety: current perspectives.. Nutrition.

[8] Batterham RL, Cowley MA, Small CJ, Herzog H, Cohen MA (2002). Gut hormone PYY(3–36) physiologically inhibits food intake.. Nature.

[9] Abbott CR, Monteiro M, Small CJ, Sajedi A, Smith KL (2005). The inhibitory effects of peripheral administration of peptide YY(3–36) and glucagon-like peptide-1 on food intake are attenuated by ablation of the vagal-brainstem-hypothalamic pathway.. Brain Res.

[10] Smith GP, Jerome C, Cushin BJ, Eterno R, Simansky KJ (1981). Abdominal vagotomy blocks the satiety effect of cholecystokinin in the rat.. Science.

[11] Koda S, Date Y, Murakami N, Shimbara T, Hanada T (2005). The role of the vagal nerve in peripheral PYY3-36-induced feeding reduction in rats.. Endocrinology.

[12] Delaere F, Magnan C, Mithieux G (2010). Hypothalamic integration of portal glucose signals and control of food intake and insulin sensitivity.. Diabetes Metab.

[13] Elmquist J, Zigman J, Lutter M (2006). Molecular determinants of energy homeostasis.. Am J Psychiatry.

[14] Liu H, Kishi T, Roseberry AG, Cai X, Lee CE (2003). Transgenic mice expressing green fluorescent protein under the control of the melanocortin-4 receptor promoter.. J Neurosci.

[15] Gantz I, Miwa H, Konda Y, Shimoto Y, Tashiro T (1993). Molecular cloning, expression, and gene localization of a fourth melanocortin receptor.. J Biol Chem.

[16] Huszar D, Lynch CA, Fairchild-Huntress V, Dunmore JH, Fang Q (1997). Targeted disruption of the melanocortin-4 receptor results in obesity in mice.. Cell.

[17] Vaisse C, Clement K, Durand E, Hercberg S, Guy-Grand B (2000). Melanocortin-4 receptor mutations are a frequent and heterogeneous cause of morbid obesity.. J Clin Invest.

[18] Yang YK, Ollmann MM, Wilson BD, Dickinson C, Yamada T (1997). Effects of recombinant agouti-signaling protein on melanocortin action.. Mol Endocrinol.

[19] Kraegen EW, Clark PW, Jenkins AB, Daley EA, Chisholm DJ (1991). Development of muscle insulin resistance after liver insulin resistance in high-fat-fed rats.. Diabetes.

[20] Mithieux G, Guignot L, Bordet JC, Wiernsperger N (2002). Intrahepatic mechanisms underlying the effect of metformin in decreasing basal glucose production in rats fed a high-fat diet.. Diabetes.

[21] Booth DA, Lovett D, Simson PC (1970). Subcutaneous dialysis in the study of the effects of nutrients on feeding.. Physiol Behav.

[22] Rolls BJ, Hetherington M, Burley VJ (1988). The specificity of satiety: the influence of foods of different macronutrient content on the development of satiety.. Physiol Behav.

[23] Gannon MC, Nuttall FQ (2004). Effect of a high-protein, low-carbohydrate diet on blood glucose control in people with type 2 diabetes.. Diabetes.

[24] Gout J, Sarafian D, Tirard J, Blondet A, Vigier M (2008). Leptin infusion and obesity in mouse cause alterations in the hypothalamic melanocortin system.. Obesity.

[25] Gout J, Sarafian D, Mutel E, Vigier M, Rajas F (2010). Metabolic and melanocortin gene expression alterations in male offspring of obese mice.. Mol Cell Endocrinol.

[26] Balthasar N, Dalgaard LT, Lee CE, Yu J, Funahashi H (2005). Divergence of melanocortin pathways in the control of food intake and energy expenditure.. Cell.

[27] Challis BG, Coll AP, Yeo GS, Pinnock SB, Dickson SL (2004). Mice lacking pro-opiomelanocortin are sensitive to high-fat feeding but respond normally to the acute anorectic effects of peptide-YY(3–36).. Proc Natl Acad Sci U S A.

[28] Wang H, Storlien LH, Huang XF (2002). Effects of dietary fat types on body fatness, leptin, and ARC leptin receptor, NPY, and AgRP mRNA expression.. Am J Physiol Endocrinol Metab.

[29] Archer ZA, Corneloup J, Rayner DV, Barrett P, Moar KM (2007). Solid and liquid obesogenic diets induce obe- sity and counter-regulatory changes in hypothalamic gene expression in juvenile Sprague-Dawley rats.. J Nutr.

[30] Barkeling B, Rossner S, Bjorvell H (1990). Effects of a high-protein meal (meat) and a high-carbohydrate meal (vegetarian) on satiety measured by automated computerized monitoring of subsequent food intake, motivation to eat and food preferences.. Int J Obes.

[31] Mithieux G (2005). The new functions of the gut in the control of glucose homeostasis.. Curr Opin Clin Nutr Metab Care.

[32] Pillot B, Soty M, Gautier-Stein A, Zitoun C, Mithieux G (2009). Protein feeding promotes redistribution of endogenous glucose production to the kidney and potentiates its suppression by insulin.. Endocrinology.

[33] Mithieux G, Misery P, Magnan C, Pillot B, Gautier-Stein A (2005). Portal sensing of intestinal gluconeogenesis is a mechanistic link in the diminution of food intake induced by diet protein.. Cell Metab.

[34] Luquet S, Perez FA, Hnasko TS, Palmiter RD (2005). NPY/AgRP neurons are essential for feeding in adult mice but can be ablated in neonates.. Science.

[35] Luquet S, Phillips CT, Palmiter RD (2007). NPY/AgRP neurons are not essential for feeding responses to glucoprivation.. Peptides.

[36] Irani BG, Xiang Z, Yarandi HN, Holder JR, Moore MC (2011). Implication of the melanocortin-3 receptor in the regulation of food intake.. Eur J Pharmacol.

[37] Benoit SC, Air EL, Coolen LM, Strauss R, Jackman A (2002). The catabolic action of insulin in the brain is mediated by melanocortins.. J Neurosci.

